# The Chemokine CXCL16 Is a New Biomarker for Lymph Node Analysis of Colon Cancer Outcome

**DOI:** 10.3390/ijms20225793

**Published:** 2019-11-18

**Authors:** Manar AbdelMageed, Haytham Ali, Lina Olsson, Gudrun Lindmark, Marie-Louise Hammarström, Sten Hammarström, Basel Sitohy

**Affiliations:** 1 Department of Clinical Microbiology, Infection and Immunology, Umeå University, SE-90185 Umeå, Sweden; manar.abdelmageed@umu.se (M.A.); haythamaa@gmail.com (H.A.); lina.olsson@umu.se (L.O.); marie-louise.hammarstrom@umu.se (M.-L.H.); sten.hammarstrom@umu.se (S.H.); 2 Department of Radiation Sciences, Oncology, Umeå University, SE-90185 Umeå, Sweden; 3 Department of Pathology, Faculty of Veterinary Medicine, Zagazig University, Zagazig 44511, Egypt; 4 Institution of Clinical Sciences, Lund University, SE-25187 Helsingborg, Sweden; Lindmarkgudrun@gmail.com

**Keywords:** chemokines, CXCL17, CEA, EpCAM, qRT-PCR, immunohistochemistry, immunomorphometry

## Abstract

Chemokines are important in the development and progression of tumors. We investigated the expression of CXCL14 and CXCL16 in colon cancer. Expression of mRNA was assessed in primary tumors and lymph nodes and CXCL16 mRNA levels were correlated to patient’s survival. Protein expression was investigated by two-color immunofluorescence and immunomorphometry. CXCL14 and CXCL16 mRNA levels and protein expression were significantly higher in colon cancer primary tumors compared to apparently normal colon tissue. Positive cells were tumor cells, as revealed by anti-CEA and anti-EpCAM staining. CXCL16, but not CXCL14, mRNA levels were significantly higher in hematoxylin and eosin positive (H&E(+)) compared to H&E(−) colon cancer lymph nodes or control nodes (*P* < 0.0001). CXCL16 mRNA was expressed in 5/5 colon cancer cell lines while CXCL14 was expressed significantly in only one. Kaplan-Meier analysis revealed that colon cancer patients with lymph nodes expressing high or very high levels (7.2 and 11.4 copies/18S rRNA unit, respectively) of CXCL16 mRNA had a decreased mean survival time of 30 and 46 months at the 12-year follow-up (*P* = 0.04, *P* = 0.005, respectively). In conclusion, high expression of CXCL16 mRNA in regional lymph nodes of colon cancer patients is a sign of a poor prognosis.

## 1. Introduction

Chemokines constitute a superfamily of chemotactic cytokines that bind to receptors belonging to class A G-protein-coupled receptors, which trigger signaling cascades and promote multiple cellular functions [1]. Apart from recruiting leukocytes to the site of inflammation, chemokines are involved in the growth and progression of many tumor types [2]. Recently, we showed that CXCL17 is ectopically expressed in tumor cells and in metastatic tumors of regional lymph nodes of colon cancer (CC) patients [3]. CXCL17 was found to be a marker for poor prognosis of CC and particularly useful for detecting less differentiated tumors expressing relatively low carcinoembryonic antigen (CEA) mRNA levels [3,4]. Inspired by the positive findings for CXCL17, we decided to investigate whether other chemokines of a CXCL-type would add clinically useful information in CC. CXCL14 and CXCL16 were chosen.

CXCL14 exhibits multiple functions, including an inhibitory effect on angiogenesis and a stimulatory effect on chemotaxis of several types of immune cells, including natural killer cells, B-cells, macrophages, monocytes, and immature dendritic cells [5]. Its receptor is still unknown. In colorectal cancer (CRC), conflicting results have been reported: one group demonstrated that high levels of CXCL14 were correlated with a poor prognosis [6], while two groups reported the opposite result [5,7].

CXCL16 exists in two forms: a transmembrane form and a soluble form (sCXCL16). The latter is released from the membrane by proteolytic enzymes [8,9]. The transmembrane form of CXCL16 can function as a cell adhesion molecule for cells expressing its receptor CXCR6, e.g., activated CD8 T cells and natural killer T cells (NKT cells), whereas sCXCL16 is a chemoattractant for CXCR6-expressing cells [10,11]. Strong expression of the CXCL16 protein in primary CRC tumor tissue was reported to be correlated to high numbers of tumor infiltrating T lymphocytes and a good prognosis [12]. Conversely, high levels of soluble CXCL16 in preoperative serum of CRC patients were associated with a poor prognosis [13].

In this study, we investigated the expression levels of CXCL14 and CXCL16 in primary CC tumors and CC cell lines as well as regional lymph nodes. These chemokines were analyzed both at the mRNA and protein levels. However, only CXCL16 mRNA expression levels were increased in CC regional lymph nodes with metastases. Therefore, we developed a new highly specific quantitative reverse transcriptase-polymerase chain reaction (qRT-PCR) assay with an RNA copy standard for determining absolute expression levels when normalized to the level of the housekeeping gene 18S rRNA and used it to analyze mesenteric lymph nodes of CC patients. CXCL16 mRNA analysis of lymph nodes was shown to give valuable prognostic information about patient survival in CC after curative surgery, either by itself or in combination with analysis of the tumor marker CEA mRNA.

## 2. Results

### 2.1. mRNA Levels of Chemokines CXCL14 and CXCL16 in Primary Colon Tumors and Colon Carcinoma Cell Lines

**Figure 1:**
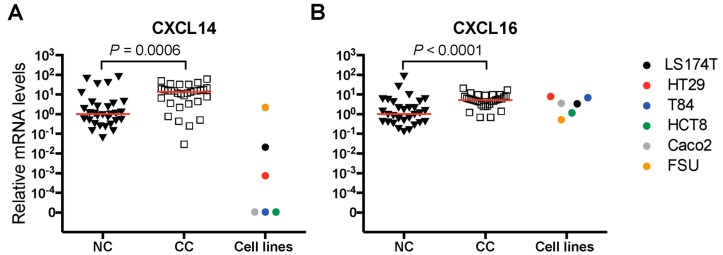
Relative mRNA levels of CXCL14 (**A**) and CXCL16 (**B**) in primary colon cancer tissues (CC) compared to adjacent normal colon margins (NC). Relative mRNA levels in the 5 CC cell lines; LS174T, HT29, T84, HCT8 and Caco2, and primary foreskin fibroblast cells (FSU) is also depicted (Cell lines). Relative mRNA levels were calculated as described in the Materials and Methods section. Red horizontal lines indicate median values*. P*-values were calculated by two-tailed Mann-Whitney test.

The median expression levels of CXCL14 and CXCL16 mRNA in primary colon tumors (*n* = 32) were 13 and five times higher than the median expression levels in normal colon tissue (*n* = 30), respectively. Both differences were highly significant (*P* = 0.0006 and *P* < 0.0001, Figure 1A,B). No difference between different T stages of the primary tumor, i.e., T2 to T4, in CXCL14 and CXCL16 mRNA expression levels was seen. Similarly, there was no statistically significant difference in CXCL14 and CXCL16 mRNA levels between primary tumors from patients belonging to different TNM stages of I to IV. However, only two patients in this clinical trial were in stage IV.

Analysis of CXCL14 and CXCL16 mRNA levels in five CC cell lines revealed an interesting difference between the two chemokines. While CXCL16 mRNA was expressed at similar levels to those of primary CC tumors in all five cell lines (Figure 1B), CXCL14 was expressed at significant levels in only one cell line (LS174T). However, the expression level was 650 times lower than in CC primary tumors (Figure 1A). Notably, both chemokine mRNAs were expressed in the primary foreskin fibroblast cell line (FSU) (Figure 1A,B).

CXCL17 mRNA expression has been previously determined in all 32 primary tumors in this study [3,4]. The correlations between the mRNA expression levels of CXCL14, CXCL16, and CXCL17 in the primary tumor tissues were assessed by a two-tailed Spearman’s rank order correlation test. Significant correlations were seen between mRNA levels of CXCL14 and both CXCL16 (r = 0.34, *P* = 0.05) and CXCL17 (r = 0.34, *P* = 0.05), but not between CXCL16 and CXCL17 (r = 0.29, *P* = 0.1).

### 2.2. mRNA Levels of Chemokines CXCL14 and CXCL16 in Regional Lymph Nodes of CC Patients

**Figure 2:**
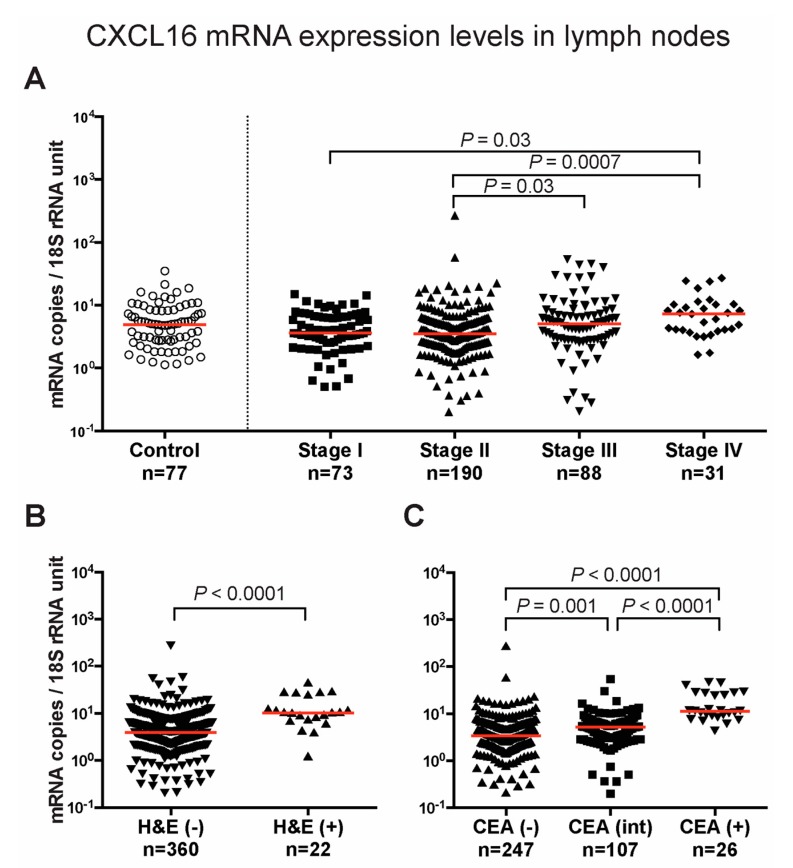
CXCL16 mRNA expression levels in lymph nodes. (**A**) CXCL16 mRNA levels in lymph nodes from non-cancerous disease patients (Control) and colon cancer patients in different TNM stages (Stage I–IV). (**B**) CXCL16 mRNA levels in non-metastatic (H&E(−)) and metastatic (H&E(+)) lymph nodes. In (**C**) lymph nodes were divided into three groups according to their CEA mRNA levels; CEA(−) = CEA mRNA levels < 0.013 copies/18S rRNA unit, CEA(int) = intermediate CEA mRNA levels, that is 0.013–3.67 copies/18S rRNA unit, and CEA(+) = CEA mRNA levels > 3.67 copies/18S rRNA unit. Red horizontal lines indicate median values. *n* = number of lymph nodes. *P*-values were calculated by Kruskal–Wallis non-parametric ANOVA followed by post hoc Dunn’s test for multiple comparisons in (**A**,**C**) and by two-tailed Mann–Whitney test for comparison between expression levels in (**B**).

The relative expression levels of CXCL14 and CXCL16 mRNA were determined in a panel of 30 regional lymph nodes from 28 CC patients and 10 lymph nodes from 10 control patients (Supplementary Figure S1). Hematoxylin and eosin positive nodes [H&E(+)] along with H&E(−) lymph nodes are shown separately. While there was a significant difference between H&E(+) lymph nodes and H&E(−) lymph nodes and controls for CXCL16 mRNA (*P* = 0.05 and *P* = 0.02, respectively), no difference between the three lymph node groups was seen for CXCL14. These results suggest that CXCL16 mRNA expression, in contrast to CXCL14 mRNA, may act as a biomarker for poor prognosis. Therefore, a specific qRT-PCR assay with an RNA copy standard was constructed for accurate determination of CXCL16 mRNA levels and was tested on 382 lymph nodes from 121 CC patients representing all four TNM clinical stages (Figure 2A). The median values of expression were 3.6, 3.5, 5.1, and 7.3 copies/18S rRNA unit in stages I, II, III, and IV, respectively. The difference in CXCL16 mRNA expression levels was significant between stages I and IV (*P* = 0.03) and II and IV (*P* = 0.0007) as well as between stages II and III (*P* = 0.03), as determined by Dunn’s multiple comparison test (Figure 2A). Twenty-two of the lymph nodes were H&E(+) and 360 were H&E(−). The CXCL16 mRNA levels were significantly higher (*P* < 0.0001) in the H&E(+) than the H&E(−) lymph nodes with median values of 10.3 and 3.9 copies/18S rRNA unit, respectively (Figure 2B). These lymph nodes were divided into three groups with respect to CEA expression levels: CEA(−), mRNA values at or below the background level (<0.013 mRNA copies/18S rRNA unit), CEA(int), CEA mRNA values (0.013–3.67 mRNA copies/18S rRNA unit), and CEA(+) with mRNA values above the clinical cut-off level (>3.67 mRNA copies/18S rRNA unit). The expression levels of CXCL16 varied significantly (*P* < 0.0001) between the different CEA groups. The median values of CXCL16 mRNA were 11.4, 5.2, and 3.4 mRNA copies/18S rRNA unit in the CEA(+), CEA(int), and CEA(−) groups, respectively (Figure 2C). The difference between the CEA(+) group and each of the CEA(int) and CEA(−) groups was highly significant (*P* < 0.0001).

### 2.3. Correlation between Expression Levels of CXCL16 mRNA, CEA mRNA, and CXCL17 mRNA in Lymph Nodes of CC Patients

**Figure 3:**
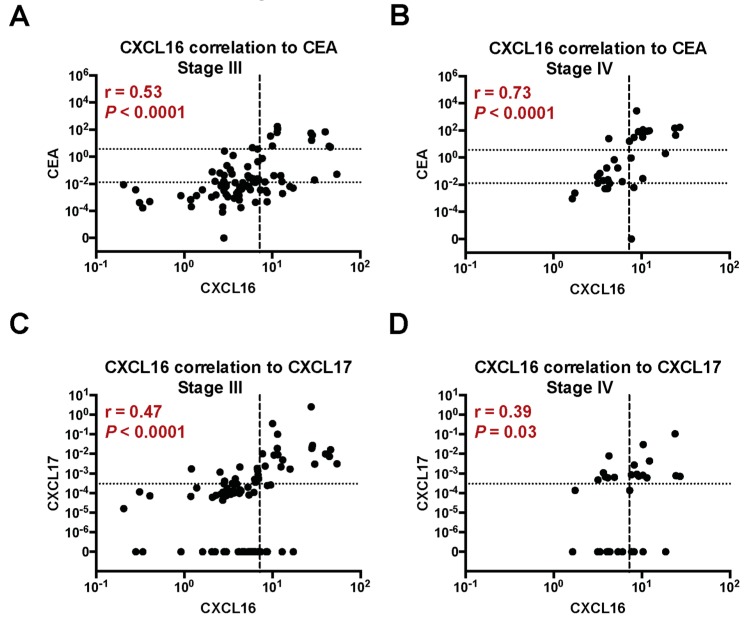
Correlations between mRNA levels of CXCL16 and mRNA levels of CEA and CXCL17 in lymph nodes of CC patients in TNM stages III and IV. (**A**,**B**) The correlations between CXCL16 mRNA levels and mRNA levels of CEA in patients in stages III and IV. (**C**,**D**) The correlations between CXCL16 mRNA levels and mRNA levels of CXCL17 in patients in stages III and IV. Dashed lines indicate a clinical cut-off value of 7.2 mRNA copies/18S rRNA unit of CXCL16. Dotted lines in (**A**,**B**) indicate the borders between three groups of lymph nodes divided according to their CEA mRNA levels; CEA(*−*) = CEA mRNA levels < 0.013 copies/18S rRNA unit, CEA(int) = intermediate CEA mRNA levels, that is 0.013–3.67 copies/18S rRNA unit, and CEA(+) = CEA mRNA levels > 3.67 copies/18S rRNA unit. Dotted lines in (**C**,**D**) indicate the border between CXCL17(–) and CXCL17(+) lymph nodes, that is, a CXCL17 mRNA level of 0.0003 mRNA copies/18S rRNA unit. The correlation coefficients (r) and the P-values were calculated by two-tailed Spearman’s rank order correlation test. Levels are given as mRNA copies/18S rRNA unit.

CEA and CXCL17 mRNA expression has been previously determined in 32 primary tumors and in 382 of the lymph nodes analyzed in this study [3,4]. The correlations between CXCL16 and both CEA and CXCL17 mRNA expression in all lymph nodes of CC patients were highly significant (r = 0.32, *P* < 0.0001 for CEA, and r = 0.36, *P* < 0.0001 for CXCL17) (data not shown). The correlations were strongest for stage III and IV patients (r = 0.53, *P* < 0.0001, r = 0.73, *P* < 0.0001, respectively, for CEA and r = 0.47, *P* < 0.0001, r = 0.39, *P* = 0.03, respectively, for CXCL17), as shown in Figure 3A–D.

### 2.4. CXCL16 mRNA Expression in Regional Lymph Nodes can Predict Disease Recurrence

**Figure 4:**
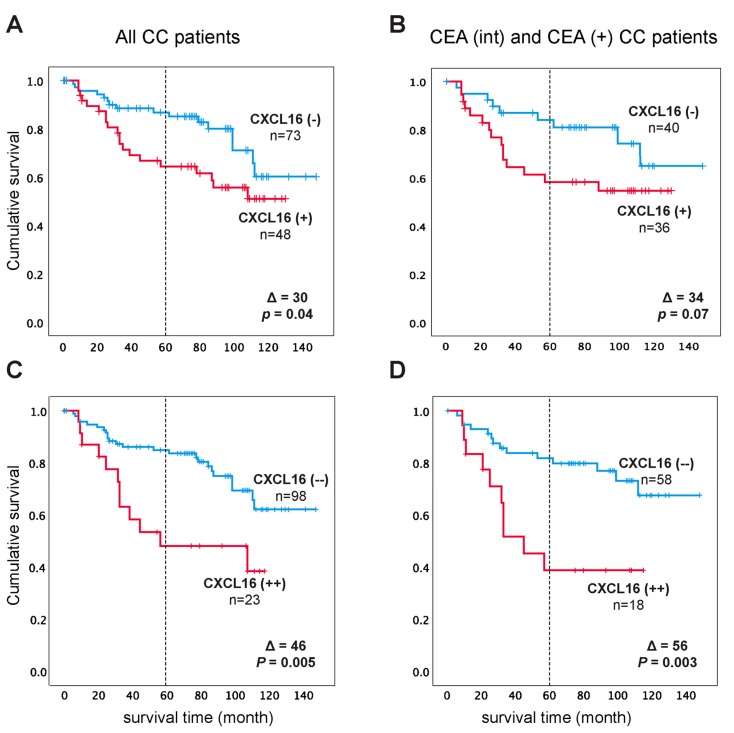
(**A**,**B**) Kaplan–Meier cumulative survival curves for CC patients divided into two groups CXCL16(−) and CXCL16(+) according to the median of mRNA expression in the highest lymph nodes in the CC patients in TNM stages III and IV (7.2 mRNA copies/ 18S rRNA unit). In (**B**) the Kaplan–Meier cumulative survival curves for CXCL16(-) and CXCL16(+) patients are restricted to the CEA(+) plus CEA(int) subgroup of CC patients. (**C**,**D**) Kaplan–Meier cumulative survival curves for CC patients divided into two groups CXCL16(--) and CXCL16(++) according to the median of mRNA expression in lymph nodes from CC patients in the CEA(+) group (11.4 mRNA copies/ 18S rRNA unit). In (**D**) the Kaplan–Meier cumulative survival curves for CXCL16(--) and CXCL16(++) patients are restricted to the CEA(+) plus CEA(int) subgroup of CC patients. The patients were followed for 12 years. Differences in disease-free survival time after surgery between the two groups are given as a ∆-value in months and statistical significance as P-values. n = number of patients in the respective group.

To investigate how determining CXCL16 mRNA expression levels in lymph nodes could be clinically relevant for predicting tumor recurrence after surgery, we calculated the hazard risk ratio using Cox regression analysis. Each patient was represented by the lymph node with the highest level of expression. The patients were divided into two groups according to the median of the expression level in the highest lymph nodes of the CC patients in stages III and IV, which was 7.2 mRNA copies/18S rRNA unit. Patients in the high expression group [CXCL16(+) group, *n* = 48] showed a 2.4-fold increased recurrence rate compared to the low expression group [CXCL16(−) group, *n* = 73] when followed for five years and 2.0-fold at a follow-up time of 12 years (*P* = 0.013 and *P* = 0.045, respectively) and a decreased mean survival time of eight months five years after surgery and 30 months 12 years after surgery (*P* = 0.010 and *P* = 0.041, respectively), according to Kaplan-Meier analysis (Figure 4A and Table 1). If the analysis was restricted to CC patients with CEA levels above the control level, which are the patients in the CEA(int) and CEA(+) groups combined, the recurrence rate was a 2.2-fold higher trend but not significant in the CXCL16(+) group when followed for 5 years and 2.1-fold when followed for up to 12 years (*P* = 0.067 and *P* = 0.082, respectively) with decreased mean survival time by eight months in five years and by 34 months in 12 years (*P* = 0.060, *P* = 0.075, respectively) when compared to the CXCL16(−) group (Figure 4B and Table 1). Therefore, using the 7.2 copies/18S rRNA cut-off level for CXCL16 mRNA and restricting analysis to patients with positive CEA mRNA values does not add prognostic information compared to analysis of the entire patient group.

Interestingly, however, if the cut-off level of CXCL16 mRNA was adjusted to 11.4 copies/18S rRNA unit (corresponding to the median value of the CEA(+) group), increased discrimination between the positive group [CXCl16(++) group] and the negative group [CXCl16(−−) group] was obtained. When all CC patients were included in the analysis, the recurrence rate increased to 2.5 times both at five years and at 12 years of observation (*P* = 0.01 and *P* = 0.007, respectively) and the mean survival time decreased by 8 months at five years and 46 months at 12 years (*P* = 0.01 and *P* = 0.005, respectively) (Figure 4C and Table 1). Moreover, if the group of patients subjected to analysis only included those that had a positive CEA mRNA value, e.g., the CEA(int) plus CEA(+) group and using the CXCL16 mRNA cut-off level of 11.4, the recurrence rate was further increased to 2.9 folds at five years and 3.2-fold at 12 years *(P* = 0.01 and *P* = 0.005, respectively) and mean survival time decreased by 11 months at five years and 56 months at 12 years (*P* = 0.008 and *P* = 0.003, respectively) when compared to the CXCL16(−−) group (Figure 4D and Table 1). These results demonstrate that CEA mRNA analysis adds prognostic information to CXCL16 mRNA analysis if the highest cut-off level is used.

In contrast, if Kaplan-Meier analysis was restricted to CC patients with CXCL17 levels higher than the clinical cut-off (i.e., 0.0003 CXCL17 mRNA copies/18S rRNA unit), the difference in the recurrence rate between the two CXCL16 mRNA groups (using the 7.2 copies/18S rRNA cut-off level) increased 1.7-fold at five years and 1.8-fold at 12 years (*P* = 0.22 and *P* = 0.21, respectively). The difference in mean survival time between the two groups was eight months at five years (*P* = 0.21) and 16 months at the 12-year follow-up (*P* = 0.19). Data not shown. None of these differences were statistically significant.

### 2.5. Expression of CXCL14 and CXCL16 Proteins in CC Tumors and Normal Colon Tissue and Lymph Nodes as Determined by IHC and IF

**Figure 5:**
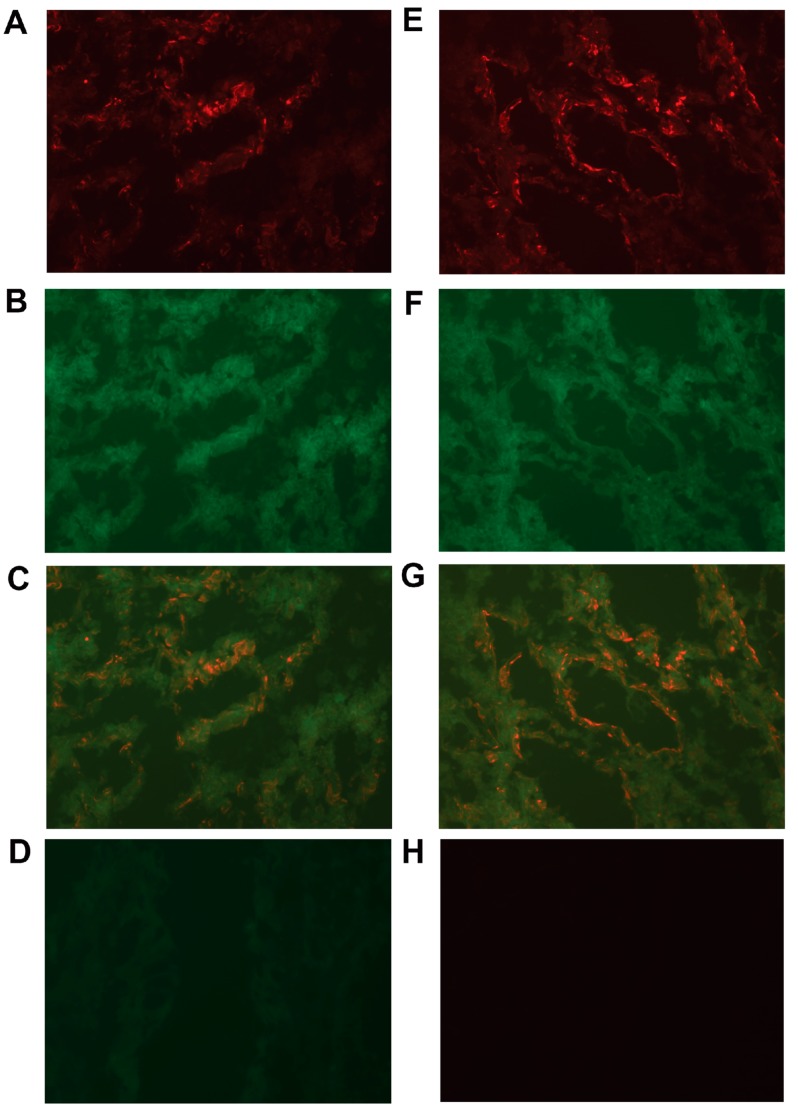
Two-color immunofluorescence staining of primary colon cancer tissue with anti-CXCL14 and BerEP4, and anti-CXCL16 and BerEP4. (**A**) Anti-CXCL14, (**E**) Anti-CXCL16 both red color. (**B**,**F**) BerEP4 mAb, green color. (**C**,**G**) Overlays giving yellow color of double-stained areas. (**D**) FITC-conjugated mouse IgG; negative control for BerEP4. (**H**) Rabbit IgG; negative control for anti-CXCL14 and anti-CXCL16. Original magnification: ×200.

To investigate whether primary CC tumors expressed CXCL14 and/or CXCL16 proteins, we performed two-color immunofluorescence experiments using anti-CXCL14 and anti-CXCL16 antibodies in combination with the anti-epithelial cell mAb BerEP4. As can be seen in Figure 5, the overlay pictures (Figure 5C,G) demonstrate that many primary CC tumor cells expressed CXCL14 and CXCL16.

**Figure 6:**
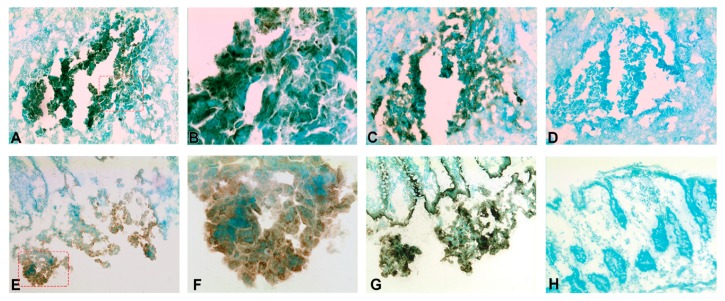
Immunoperoxidase staining of frozen sections of primary colon cancer tissue. (**A**) Anti-CXCL14 staining of primary CC tissue, original magnification ×100. (**B**) Higher magnification of indicated area in (**A**), ×400. (**C**) Anti-CEA staining of primary CC tissue, original magnification X100. (**D**) Negative control of a primary CC tissue, original magnification ×100. (**E**) Anti-CXCL16 staining of primary CC tissue, original magnification ×100. (**F**) Higher magnification of indicated area in (**E**), ×400. (**G**) Anti-CEA staining of primary CC tissue, original magnification ×400. (**H**) Anti-CXCL16 staining of normal colon tissue, original magnification ×100. Note that (**A**), (**C**) and (**D**), and (**E**) and (**G**) are consecutive sections.

**Figure 7:**
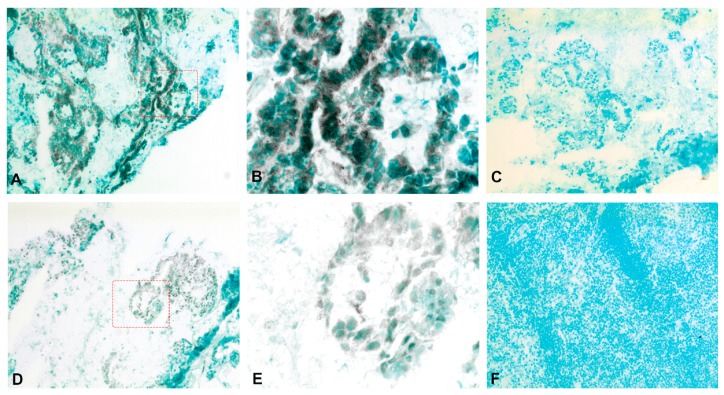
Immunoperoxidase staining of frozen lymph node tissue sections with metastatic colon cancer. (**A**) Anti-CXCL14 staining of an H&E(+) lymph node of a CC patient, original magnification ×100. (**B**) Higher magnification of indicated area in (A), ×400. (**C**) Negative control of H&E(+) lymph node of a CC patient, original magnification ×100. (**D**) Anti-CXCL16 staining of an H&E(+) lymph node of a CC patient, original magnification ×100. (**E**) Higher magnification of indicated area in (**D**), ×400. (**F**) Anti-CXCL16 staining of an H&E(−) lymph node of a CC patient, original magnification ×100.

Figure 6 shows the result of consecutive-section-staining of primary CC tumors with anti-CXCL14 and anti-CEA and anti-CXCL16 and anti-CEA, respectively. Many tumor cells are stained by both markers. Anti-CXCL16 staining appears to be stronger than the anti-CXCL14 staining. Both anti-CXCL14 and anti-CXCL16 stained groups of cells in H&E(+) lymph nodes (Figure 7).

**Figure 8:**
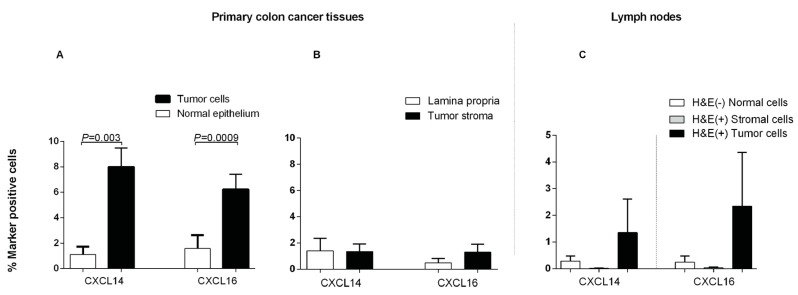
Frequencies of CXCL14 and CXCL16 positive stained cells in primary colon cancer tissue (CC) compared to normal colon tissue and in H&E(+) and H&E(-) lymph nodes from CC patients as determined by immunomorphometric analysis according to Weibel (1979). (**A**) CC tumor cells (black bars) compared with normal epithelial cells (open bars). (**B**) CC tumor stroma (black bars) compared with lamina propria in normal colon (open bars). (**C**) Metastatic tumors cells (black bars) and other cells of H&E(+) lymph nodes (grey bars) compared to cells of H&E(−) lymph nodes (open bars). Bars represent mean +1 SEM. *P*-values for comparison between tumor and normal tissue by two-sided Mann–Whitney t-test are given. Ten primary CC tumors and 10 normal colon tissue samples were analyzed in A and B. Twelve lymph nodes were analyzed in C.

Weibel counting revealed that both CXCL14 and CXCL16 protein positive cells were present at significantly higher numbers in primary CC tumor cells compared to normal colon epithelium (*P =* 0.003 and 0.0009, respectively. Figure 8A). No difference was seen when tumor stroma was compared with lamina propria of normal colon mucosa (Figure 8B). Assessing the protein expression of both CXCL14 and CXCL16 in lymph nodes, CC patients showed no significant differences between tumor cells *(P* = 0.9 and *P* > 0.9) and other cells in H&E(+) lymph nodes when compared to cells in H&E(−) lymph nodes (*P* = 0.9 and *P* = 0.9), which is likely due to the small number of lymph nodes that could be obtained for this part of the study (Figure 8C).

## 3. Discussion

In this study, we assessed the expression level of CXCL16 and CXCL14 in CC primary tumors, lymph nodes, and cell lines revealing that CXCL16 mRNA expression is an excellent biomarker to predict disease recurrence if used for analysis of lymph nodes. CXCL16 was expressed at increased levels in primary tumors and CC cell lines compared to apparently normal colon tissue both at the mRNA and protein levels. Moreover, high levels of CXCL16 mRNA in regional lymph nodes obtained at surgery of CC patients predicted poor survival. In fact, 12 years after surgery, CC patients with high levels of CXCL16 mRNA had 30 to 46 months shorter mean survival time than those with low CXCL16 mRNA levels depending on which cut-off level for CXCL16 was selected. This result contrasts with that of Hojo et al. [12], who reported that high CXCL16 levels were associated with a good prognosis. The reason for this difference is unclear. However, the two studies differ in two major ways. First, they studied primary tumors and we studied regional lymph nodes and, second, they determined protein expression by immunohistochemistry and we investigated mRNA by qRT-PCR with a copy standard. In contrast, our finding is in line with those of Matsushita et al., who studied CXCL16 in serum [13].

It is interesting to note that an increased difference in mean survival time and recurrence risk between lymph nodes from CC patients with high CXCL16 mRNA levels and those with low CXCL16 mRNA levels was achieved by the additional analysis of mRNA for the biomarker CEA. Thus, if the patients who express CEA mRNA above the background level were exclusively investigated, the difference between the high and low CXCL16 groups was relatively increased than when considering the entire CC patient group. This additional effect is, however, only noticed at the highest CXCL16 mRNA cut-off level. This effect is not seen if CXCL16 is combined with CXCL17.

Analysis of CXCL14 at the mRNA and protein levels revealed its increased expression in primary tumor tissue compared to normal colon tissues but not in four of five CC tumor cell lines. However, it was highly expressed in the fibroblast cell line (FSU) and at low but significant levels in the fifth CC cell line (LS174T), likely due to fibroblasts known to occur in this cell line [14]. It is noteworthy that CXCL14 was reported to be frequently methylated in human CC cell lines, which could explain lack of expression in several CC cell lines in this study [7]. CXCL14 mRNA was not expressed at significantly changed levels in H&E(+) lymph nodes compared to H&E(−) nodes, which indicates that CXCL14 mRNA does not seem to be of value as a prognostic lymph node marker for patients with CC. However, a recent study by Liu et al. demonstrated that CXCL14 expression was a prognostic marker in CC during the analysis of primary tumors [15].

The two-color immunofluorescence staining with anti-CXCL16 and anti-EpCAM mAb and consecutive-sections-staining procedure with anti-CEA and anti-CXCL16 in the primary tumor tissues and lymph node metastasis suggest that CXCL16 is produced mainly by primary tumor cells and metastatic tumor cells. In spite of the fact that CXCL16 can attract CD8+ T cells and NKT cells, which are cells with cytolytic powers, this does not seem to hinder tumor progression.

In conclusion, this study shows that CXCL16 mRNA is a marker for poor prognosis both independently or in combination with CEA mRNA and that it merits further studies.

## 4. Materials and Methods

### 4.1. Patients and Tissue Specimens for mRNA Analysis

Primary tumor specimens were retrieved from 32 CC patients (13 men and 19 women, median age 72 years, range 43–86 years) after surgery. None of the patients received treatment before surgery. Twelve patients were in stage I (T1-2N0M0), 10 patients were in stage II (T3-4N0M0), eight patients were in stage III (anyTN1-2M0), and two patients were in stage IV (anyTanyNM1). Primary tumor stage distribution (pT1-pT4) was 2, 10, 10, and 10, respectively. The tumor samples, approximately 0.5 × 0.5 × 0.5 cm in size, were collected immediately after resection, snap-frozen, and stored at −70 °C until undergoing RNA extraction. Normal colon samples retrieved from the proximal or distal resection margin of CC tumors were also collected from 30 patients (13 women and 17 men, median age 72 years, range 57–85 years) and treated the same way.

Lymph nodes were from 121 CC patients (median age 70 years, range 41–88 years, 66 women and 55 men). Seventy-three lymph nodes were from 23 patients in stage I, 190 lymph nodes were from 52 patients in stage II, 88 lymph nodes were from 37 patients in stage III, and 31 lymph nodes were from nine patients in stage IV. Twenty-two lymph nodes were H&E(+) and 360 lymph nodes were H&E(−). Control lymph nodes were from 13 patients (median age 23 years, range 9–32 years: three women and 10 men). Eleven patients had ulcerative colitis, one had Crohn’s disease and one had lipoma. In total, 459 lymph nodes were investigated in this analysis.

### 4.2. Patients and Tissue Specimens for Immunohistochemistry

Primary tumor tissue specimens from 10 CC patients (four men and six women, median age of 82 years) were studied. None of the patients received treatment before surgery. One tumor was in stage I, three tumors were in stage II, four tumors were in stage III, and two tumors were in stage IV. The localization of the tumors was caecum (three patients), ascending colon (three patients), transverse colon (two patients), and sigmoid colon (two patients). Primary tumor stage distribution (pT2–pT4) was 1, 6, and 3, respectively. Normal colon tissue specimens were also obtained from 10 CC patients (5 men and 5 women, median age 62 years). The tissue was taken at a distance to any macroscopically detectable lesions. The localization of the normal colonic specimens was the caecum (two patients), the ascending colon (two patients), the transverse colon (one patient), and the sigmoid colon (five patients).

Lymph nodes were from 10 patients (median age 80 years, range 71–91 years). Six lymph nodes were non-metastatic, i.e., H&E(−). These nodes were from one patient in stage I, two patients in stage II, and two patients in stage III. Seven lymph nodes were metastatic, i.e., H&E(+), from five patients in stage III.

### 4.3. Cell Lines

Five human CC cell lines (LS174T, HT29, T84, HCT8 and Caco2) and primary foreskin fibroblast cells (FSU) were cultured and analyzed for mRNA expression, as previously described [3].

### 4.4. Real-Time qRT-PCR

Commercially available TaqMan Gene Expression Assays Hs01557413_m1 and Hs00222859_m1 (Applied Biosystems, Foster City, CA, USA) were used to determine the relative mRNA expression levels of CXCL14 and CXCL16, respectively. The qRT-PCR profile in all the mRNA assays, using Taqman EZ technology (Applied Biosystems), was 50 °C for 2 min, 60 °C for 30 min, and 95 °C for 5 min, which was followed by 45 cycles at 95 °C for 20 s and at 60 °C for 1 min (LightCycler 480 RNA master hydrolysis probes, Cat. No. 04991885001, Roche, Mannheim, Germany). Emission from the released reporter dye in the PCR reaction was measured by the ABI Prism 7700 Sequence instrument (Applied Biosystems). The amount of 18S ribosomal RNA (rRNA) was determined in each sample by real-time qRT-PCR (Applied Biosystems) for normalization of chemokine mRNA levels [16]. For relative expression, chemokine mRNA expression levels (RQ) were calculated according to the equation: 2^-(∆ct of the sample—the median ∆ct-value of the normal colon tissue samples). ∆ct is the ct-value of the chemokine mRNA minus the ct-value of 18S rRNA in the same sample [3].

For absolute quantification of CXCL16 mRNA in lymph nodes, we constructed a real time qRT-PCR assay using specific primers placed in different exons and a reporter dye-labeled probe hybridizing over the exon boundary in the amplicon and specific RNA copy standards. The assay detects both transcript variants of CXCL16 mRNA (NM_022059.3 and NM_001100812.1). The primers and probe sequences were: forward primer 5′-CCCATGGGTTCAGGAATTGAT-3′, reverse primer 5′-CCACAATCCCCGAGTAAGCA-3′ and probe 5′-CTTGATCTCAAAGAATGTGGAC-3′. The reporter dye was FAM and the quencher dye was MGB. The size of the amplicon was 71 bp. The qRT-PCR profile was 60 °C for 5 min and 95 °C for 1 min, which is followed by 45 cycles of 95 °C for 15 s and 60 °C for 1 min. RNA oligonucleotides with a sequence identical to that in the area amplified in the qRT-PCR assay was custom synthesized at Dharmacon (Lafayette, CO, USA). Serial dilutions of the RNA copy standards at concentrations from 10^3^ to 10^8^ copies per µl were included in each qRT-PCR run. Concentrations in unknown samples were determined from the standard curve and expressed as copies of mRNA per µl. The concentration of 18S rRNA was expressed as arbitrary units from a standard curve of serial dilutions of a preparation of total RNA from human peripheral blood mononuclear cells. One unit was defined as the amount of 18S rRNA in 10 pg RNA [3]. CXCL16 mRNA was expressed as copies per unit of 18S rRNA. qRT-PCR assays for CEA and CXCL17 mRNAs were described earlier [3,4].

### 4.5. Antibodies and Substrate

Anti-CXCL14 (rabbit IgG; NBP1-31398; Novus, Littleton, CO, USA), anti-CXCL16 (rabbit IgG; ab101404, Abcam, Cambridge, MA, USA), anti-CEA (mouse IgG1, clone II-7, M7072, Dako, Glostrup, Denmark) and FITC-conjugated anti-EpCAM (mouse IgG1, clone BerEP4, F0860; Dako) were used as primary antibodies. Mouse IgG, ready to use (Dako), rabbit IgG ready to use (Dako) and FITC-conjugated mouse IgG (F0313; Dako) were used as negative controls. ImmPRESS micropolymer HRP conjugated anti-rabbit IgG (MP-7401, Vector laboratories, Burlingame, CA, USA), ImmPRESS micropolymer HRP conjugated anti-mouse IgG (MP-7402, Vector laboratories), and Alexa Fluor 555-conjugated goat anti-rabbit IgG (ab150078, Abcam) were used as secondary antibodies. The peroxidase substrate used was DAB (3,3′-diaminobenzidine, Vector Laboratories).

### 4.6. Immunohistochemistry

Fresh tissue samples were rinsed with cold phosphate-buffered saline (PBS), snap-frozen in iso-pentane pre-cooled in liquid nitrogen, and stored at −70 °C. Frozen tissue was cut into 4–6 µm thick sections with a cryo-microtome (MICROM HM505E, Thermo Fisher, Waltham, MA, USA). As described elsewhere [17], the sections were fixed with 4% paraformaldehyde for 15 min before air-drying, rehydration in PBS, and immersion in PBS containing 0.03% H_2_O_2_ and 2 mM NaN_3_ at 37 °C to quench endogenous peroxidase activity. Thereafter, the sections were incubated with 0.2% bovine serum albumin in PBS, which was followed by ImmPress ready-to-use horse blocking serum (Vector Laboratories) at room temperature to block non-specific binding sites. Subsequently, the sections were incubated with primary antibodies or corresponding negative controls for 1 h at room temperature, which was followed by 1 h of incubation with ImmPRESS anti-mouse IgG or ImmPRESS anti-rabbit IgG. Bound peroxidase was revealed by incubation with 0.05% DAB and 0.03% H_2_O_2_ in 0.05 M Tris buffer (pH 7.6) at room temperature, and was counterstained with methyl green. Consecutive sections were stained with anti-CEA, anti-CXCL14, or anti-CXCL16, respectively.

### 4.7. Two-Color Immunofluorescence

Sections of tumor tissues were cut and fixed as described above, and, thereafter, incubated with rabbit polyclonal anti-CXCL14 or CXCL16 antibodies or rabbit IgG as a negative control, which was followed by the Alexa Fluor 555-conjugated goat anti-rabbit IgG (red). Subsequently, the sections were incubated with FITC-conjugated BerEP4 (green) or FITC-conjugated mouse IgG as a negative control. Double-positive cells show a yellow–orange color. The sections were then mounted with SlowFade^®^ Gold Antifade Mountant (Thermo Fisher). Microscopy was done using a Nikon fluorescence microscope and images were analyzed with NIS elements software.

### 4.8. Immunomorphometry

Quantification of cell numbers was performed by Weibel [18], who analyzed the expression of CXCL14 and CXCL16 in tumor cells and in the surrounding stroma in comparison with controls. Twenty randomly chosen ocular fields were counted for each marker in each compartment. The slides were coded to avoid personal bias. An integrating cooled color 3CCD camera (Color Chilled 3 CCD Hamamatsu CameraC5810, Hamamatsu Photonics, Hamamatsu City, Japan) was used on a standard light microscope combined with a computer image analysis system (LeicaQWin, Leica Imaging Systems, Cambridge, UK) with an interactive, computerized morphometry program. Microscopic fields were selected randomly using a 40× objective and transferred to the screen, onto which a regular lattice was superimposed. Points outside the concerned tissue compartment and empty spaces were not included in the calculation. Positive cells located in the coarse points were counted, and the ratio between the number of points covering positive cells and the total number of points covering cells in the tissue under investigation was calculated for each microscopic field.

### 4.9. Statistical Analysis

The statistical significance of differences in mRNA levels and number of cells expressing different chemokines in primary CC tumors compared to normal colon tissue was calculated using the two-tailed Mann–Whitney rank sum test. Statistical significance of differences in mRNA levels between control lymph nodes and lymph nodes from different patient groups were analyzed using the Kruskal-Wallis one-way analysis of variance (ANOVA) test followed by the Dunn’s multiple comparison post-hoc test. Correlation between chemokine mRNA levels was analyzed using the non-parametric Spearman correlation coefficient. Descriptive values are expressed as mean ± standard error of the mean (SEM) for immuno-morphometric analysis and median for mRNA levels. The software utilized for statistical calculations was GraphPad Prism 6 (Graphpad Software, San Diego, CA, USA).

The SPSS software (IBM Corporation, Armonk, NY, USA) was used for statistical analyses of differences between patient groups in disease-free survival time and analyses of risk for recurrent disease after surgery, according to the Kaplan–Meier survival model in combination with the log-rank test and univariate Cox regression analysis. A *P*-value ≤ 0.05 was considered to be statistically significant.

## Figures and Tables

**Table 1:** Comparative analysis of average survival time after surgery and risk for recurrence of disease in CC patients with CXCL16(−) and CXCL16(+), and CXCL16(−−) and CXCL16(++) lymph nodes.

Patient Group	Category	5 Year Follow-Up after Surgery					12 Year Follow-Up after Surgery				
		Disease-Free Survival			Risk for Recurrence		Disease-Free Survival			Risk for Recurrence	
		Average ^a^	Difference	*P*-Value	Hazard Ratio	*P*-Value	Average ^a^	Difference	*P*-Value	Hazard Ratio	*P*-Value
		(Months)	(Months)		(95% CI) ^b^		(Months)	(Months)		(95% CI) ^b^	
All CC patients	CXCL16(−) ^c^	55					118				
	CXCL16(+)	47	8	0.01	2.4	0.013	88	30	0.041	2.0	0.045
					(1.2–4.8)					(1.0–3.8)	
	CXCL16(−−) ^d^	53					117				
	CXCL16(++)	45	8	0.01	2.5	0.013	71	46	0.005	2.6	0.007
					(1.2–5.2)					(1.3–5.2)	
CEA(int) plus CEA(+) ^e^ CC patients	CXCL16(−)	54					119				
	CXCL16(+)	46	8	0.06	2.2	0.067	85	34	0.075	2.1	0.082
					(0.9–5.0)					(0.9–4.8)	
	CXCL16(−−)	53					117				
	CXCL16(++)	42	11	0.008	2.9	0.011	61	56	0.003	3.3	0.005
					(1.3–6.5)					(1.4–7.4)	

^a^ Mean survival time after surgery for CC patients as calculated by cumulative survival analysis, according to Kaplan-Meier; ^b^ Hazard ratio with 95% confidence interval (CI) for CC patients as calculated according to univariate Cox regression analysis; ^c^ CC patients divided into two groups CXCL16(−) and CXCL16(+) according to the median value of CXCL16 mRNA expression in the highest lymph node in the CC patients in TNM stages III and IV (7.2 mRNA copies/ 18S rRNA unit); ^d^ CC patients divided into two groups CXCL16(−−) and CXCL16(++) according to the median value of CXCL16 mRNA expression in lymph nodes from CC patients in the CEA(+) group (11.4 mRNA copies/ 18S rRNA unit); ^e^ CEA(int) plus CEA(+) group: CC patients where the CEA mRNA levels in the highest lymph node is higher than the highest level of control patients lymph nodes, i.e., 0.013 mRNA copies/18S rRNA unit.

## Supplementary Materials

Supplementary materials can be found at https://www.mdpi.com/1422-0067/20/22/5793/s1.
