# Supplementary material for: The Chemokine CXCL16 Is a New Biomarker for Lymph Node Analysis of Colon Cancer Outcome
Source: Int J Mol Sci. 2019 Nov 18;20(22):5793. doi: 10.3390/ijms20225793 (PMC6888697; doi:10.3390/ijms20225793)
Supplement: Supplementary file 1 [file ijms-20-05793-s001.pdf]

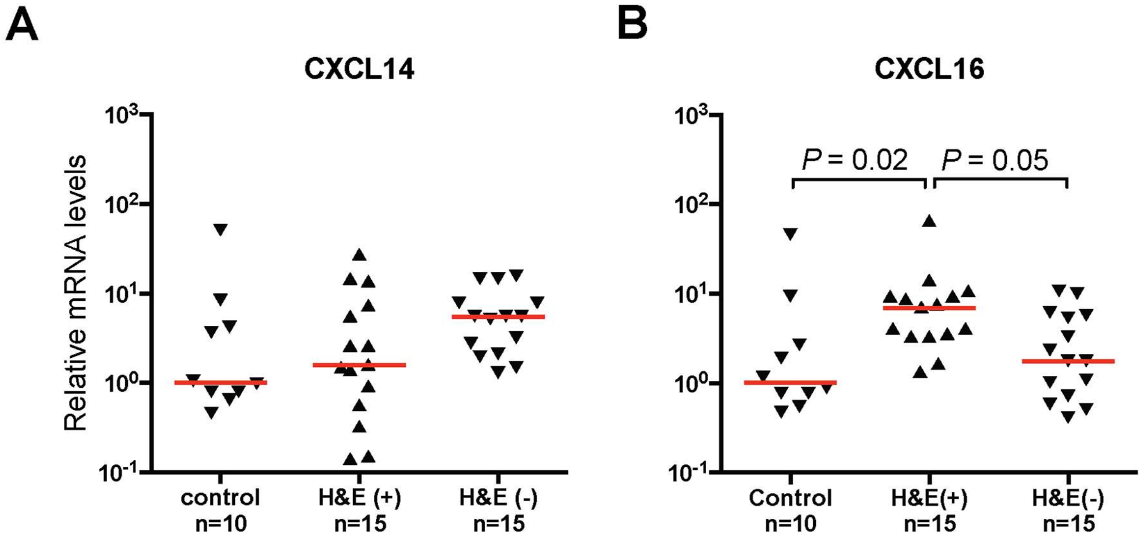

Supplementary Figure 1. Relative mRNA levels of **(A)** CXCL14 and **(B)** CXCL16 in a panel of 30 lymph nodes from 28 CC patients and 10 lymph nodes from 10 control patients. P-values were calculated by Kruskal–Wallis non-parametric ANOVA followed by a post hoc Dunn's test for multiple comparisons test. Red lines indicate median values.
